# Impact of Mental Health First Aid Training Courses on Patients' Mental Health

**DOI:** 10.1155/2022/4623869

**Published:** 2022-09-12

**Authors:** Fanli Zeng, Dexia Zhong, Xi Chen, Hongmei Li, Xiaofei Tian

**Affiliations:** Neurology Department and Nursing Department, The Seventh Affiliated Hospital, Sun Yat-sen University, Shenzhen 518107, China

## Abstract

**Background:**

With the prevalence of mental issues worldwide, more and more people are suffering from psychological torture. Mental Health Gap Action Program (mhGAP) has been introduced to improve the life quality of humans.

**Objectives:**

To explore and synthesize evidence of participants' experience of mental health first aid (MHFA) training course.

**Method:**

Peer-reviewed qualitative evidence was systematically reviewed and thematically synthesized. Cumulative Index to Nursing and Allied Health Literature (CINAHL), Medical Literature Analysis and Retrieval System Online (MEDLINE), Psychological Information (PsycINFO), PubMed, Psych ARTICLES, Web of Science, Joanna Briggs Institute (JBI), and National Institute for Health and Care Excellence (NICE) databases were searched for the inception of the present study. The study's quality was appraised using the Critical Appraisal Checklist for Qualitative Research of Joanna Briggs Institute (JBI) appraisal tool. All the participants who have attended the MHFA training course (excluding instructors) setting were included.

**Results:**

Six papers published between 2005 and 2019 were included for thematic synthesis. The review indicated that MHFA had been a positive experience for participants.

**Conclusions:**

MHFA courses can provide participants with professional knowledge of mental health counseling and improve their knowledge, practice, and attitudes towards their patients. Professional MHFA training courses should therefore be popularized and promoted among other populations.

## 1. Introduction

With the prevalence of mental issues worldwide, more and more people are suffering from psychological torture. The World Health Organization (WHO) has emphasized the importance of mental health disorders and created a Mental Health Gap Action Program (mhGAP) to improve the life quality of humans. Many people are suffering from suicide and depression [1–3]. This means that governments need to strengthen mental health to promote a better life for people worldwide. Meanwhile, a great number of people in developing countries are living with mental health disorders, which are linked to suicide, alcohol or suicide, and alcohol or drug dependence. This review aimed to review the participants' experience systematically before and after they attended the MHFA training.

There are three parts to the global concern for mental health issues [4]. It is suggested that health care workers should try to help those people with psychological disorders and provide guidance and positive influence/impact to them [5]. Mental health issues are also faced by the general population and related healthcare students [6, 7]. It has been recommended that medical students should seek help from medical schools or mental health systems [8, 9]. The Australian government has designed a Mental Health First Aid (MHFA) course to help those who are developing mental health problems or suffering a mental health crisis. The Mental Health First Aid (MHFA) program is able to help people [10, 11]. People tend to ignore mental health issues partly because they lack insight into mental health and partly because they fear the stigma that comes with mental illness [12, 13]. There were two methods in the educational process of the MHFA course which included e-learning and face-to-face learning. This review only focused on the effectiveness of the MHFA course. In a specific population, such as nurses and other medical students, they also have heavy stress in hospitals and universities [14]. Hence, it is essential to pay attention to those with mental health disorders to improve their quality of life.

The systematic review was to understand in-depth the experiences of participants who took part in the MHFA training course. Therefore, the review questions are as follows: Firstly, what is the general participants' experience of MHFA training? Secondly, what is the difference between the participants, especially healthcare students, and the other population? The types of participants, exposure, outcome measures, and studies were considered and identified by the following search strategy. Porritt et al. recommended that the PIC (population, phenomena of interest, and context) tool was much more suitable for qualitative studies [15].

## 2. Methods

This study used systematic review as an approach to answering the specific review questions. More details of participants' experiences are focused on the MHFA training than quantitative data [16]. The review study was identified with different databases, such as Cumulative Index to Nursing and Allied Health Literature (CINAHL), Medical Literature Analysis and Retrieval System Online (MEDLINE), Psychological Information (PsycINFO), PubMed, Psych ARTICLES, and Web of Science. In addition, databases of the Joanna Briggs Institute (JBI) and National Institute for Health and Care Excellence (NICE) were searched to gain more related qualitative articles. This original search terms were shown in Appendix 1. This review will include English-language articles.

### 2.1. Inclusion and Exclusion Criteria

Nine items were used to make inclusion and exclusion criteria: participants, intervention, intervention setting, study focus, outcomes, study design, time period, publication type, and language [17] (Appendix 2).

#### 2.1.1. Type of Population

This review will use the purposive sampling method to select the final population. This review only focused on the general population, excluding the instructors or facilitators without the limitation on participants' age, gender, religion, and nationality. The phenomenon of interest was participants who have had experience of MHFA training courses after completing the 12-h training or 9-h training. Participants' views of the MHFA, psychology first aid, or physical first aid were also considered.

#### 2.1.2. Type of Context and Study

The MHFA online training courses and Youth MHFA training courses were not considered because of the different teaching methods and the contents of the two-type training [16]. The studies that focused on mental health illness, issues, problems, and disorders were included, as they were relevant to the MHFA training.

The primary qualitative studies, which used grounded theory and phenomenology, were included [18]. The mixed-method literature only focused on the qualitative part to make an analysis. The meta-analysis, case studies, and randomized controlled trial (RCT) were excluded. Therefore, the review focused on qualitative studies, qualitative analysis studies, qualitative research, and mixed-methods papers and did not consider the quantitative studies. All the qualitative studies were written in English to reduce language bias.

### 2.2. Study Quality Procedures and Assessment

The systematic review needed two reviewers to analyze independently to increase the trustworthiness and decrease the personal variation [18]. The student reviewer would double-check the quality assessment and data extraction every week to ensure that the appraisal and analysis are objective and reliable.

All the included studies were collected and appraised with empirical data procedures, which was an empirical analysis to improve accuracy and estimate errors [19, 20]. This review used the corresponding checklists from the Critical Appraisal Checklist for Qualitative Research of JBI. The details of the comparison were shown in Appendix 3. The JBI checklist contained ten items and made it easier to appraise the qualitative research. Answers to the ten items were categorized as yes/no/not clear/not applicable [21]. The details of quality appraisal are shown in Appendix 4.

### 2.3. Data Extraction

Data extraction is the process of analyzing and trawling data to retrieve relevant information from data sources of a particular pattern, such as a database [22]. According to Higgins and Green [23], the Cochrane handbook for systematic reviews is a “gold standard” of interventions to guide the review process and implementation. Several data extraction tools include the CASP tool, Evaluation Tool for Qualitative Studies (ETQS), and JBI tool [24].

### 2.4. Data Synthesis

Data synthesis was an integral part of systematic reviews [22, 25]. According to the Cochrane handbook, a professional synthesis method can help the reviewer gain a deeper analysis of primary studies' findings [26].

## 3. Results

### 3.1. Results of the Research

**Figure 1:**
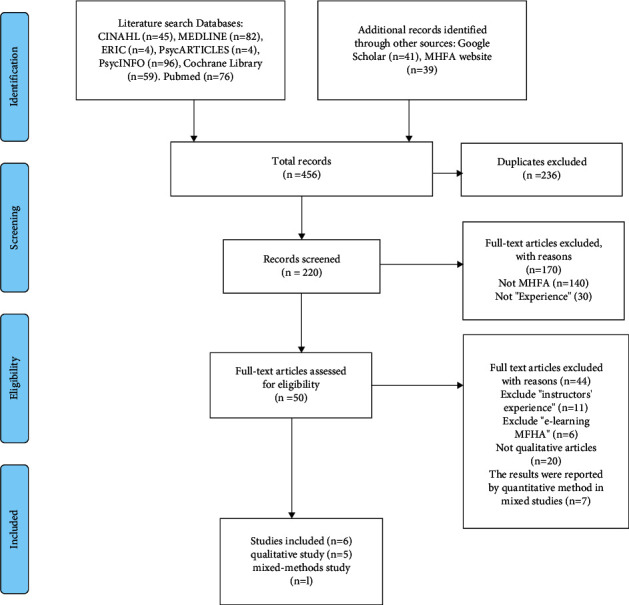
A systematic qualitative review and thematic synthesis is the participants' experiences of the mental health first aid training.

Finally, there were six studies selected, which include five qualitative studies and one mixed-methods paper for inclusion (see Figure 1). It is recommended that PRISMA could make a clear flow chart in systematic reviews and meta-analyses [27–29]. Four were interviews, one was a survey, and another was a questionnaire. These studies were published between 2005 and 2019 and included 238 participants. Three studies were from Australia, the USA, Sweden, and Hong Kong (China). Three studies considered the same sample focused on university students, especially nursing and midwifery students [30–32]. One paper had 15 instructors who had finished a 5-day instructor training with the ability to teach the MHFA-USA course to others [11]. The details are shown in Appendix 5.

### 3.2. Results of Quality Appraisal

Six studies clearly stated their objectives and focused on the different size of studies (see Appendix 6). Three of these papers were high-quality [30, 31, 33], while the other three were medium-quality papers through the quality appraisal [11, 32, 34]. As was shown in Appendix 6, the studies' congruity was high, it was worth analyzing, and these six papers were all included. Two identified analytical themes include to improve individuals' MHFA knowledge and practical ability and to improve individuals' attitude.

#### 3.2.1. Individuals' MHFA Knowledge and Practical Ability Were Improved

All six papers indicated that the MHFA training course could enhance participants' knowledge and understanding of mental health issues [11, 30–34]. Besides, some participants also highlighted that the MHFA course could improve their career confidence and provide more information resources and services in their local contexts [30]. Moreover, one participant described that the MHFA had a positive effect and benefit to “being a good listener and friend and giving helpful advice” [34]. Some participants stated the MHFA training was “very interesting,” “very helpful,” “beneficial and valuable,” and “would encourage everyone to attend it” [34]. On the other hand, the participants noticed that the instructor was “very good,” who could teach them professional knowledge of mental health [33].

The papers indicated that the MHFA course could improve participants' mental health essential skills and techniques to support patients [11, 30–32, 34]. Additionally, one of the trainees reported that the MHFA course was “great” and could teach him/her to interact with people through the ALGEE [11]. It was highlighted that the MHFA course provided trainees with practical skills to people [11].

Four studies indicated that the MHFA course could improve participants' awareness and confidence in dealing with mental health issues [11, 32, 34, 35]. It is suggested that the graduates were more willing and able to help others after joining the MHFA training, as one participant reported that it provided some “tangible tools” [11]. Similarly, Rodgers et al. indicated that some participants replied that the course gave them “more confidence” to ask patient's feelings and thoughts during offering the support [32].

It was interesting that the MHFA course not only improved awareness of other people's mental status but also improved understanding of their own mental health conditions [11, 30, 34]. Likely, some participants promoted the MHFA training to make them “more aware” for themselves including physical and mentally health [11]. The studies indicated that the MHFA training enhanced the sense of achievement and satisfaction [30, 34]. For instance, one participant perceived the MHFA course as a bridge and provided her “more confidence” in dealing with her father's situation and helping him [30].

#### 3.2.2. Improved Individuals' Attitude

The papers indicated that the MFHA course had changed participants' beliefs and values after joining the course [11, 30, 34, 35]. One person expressed that he/she sometimes was “inpatient and annoying” to take care of his/her friend before the MHFA training, and this course made them understand their friend's “negative mood or mindset” [30]. The three studies indicated that the participants became more willing to provide support to people [11, 30, 34]. A nursing student said that she was not “more willing to offer help,” as the stress of being a nurse was very high [30]. Lucksted et al. indicated that the MHFA course also increased the intention to help people, especially permission and responsibility [11]. Two studies indicated that MHFA training could reduce participants' stigma and avoidance after joining the course [11, 32]. For instance, many people feared schizophrenia. However, one participant reported that the course made him “more compassionate and a little fearless” to talk with others after the course [11]. Rodgers et al. pointed out that some participants faced challenges in supporting MHFA [32]. One respondent reported they needed to “maintain” and “keep supporting” to help, and another participant stated that he/she lacked the “energy or emotional” ability to deal with the two people at the same time.

## 4. Discussion

According to this review and thematic synthesis, participants' experiences of the MHFA course after training were categorized into two key themes: improved individuals' MHFA knowledge and practical ability and then improved individuals' attitude. The six subthemes indicated that the MHFA training course improved participators' knowledge and practical support of mental health illness [11, 30, 32, 34, 35]. Five papers indicated that the instructors teach them the first aid actions to offer immediately when someone needs help [11, 30–32, 34]. In terms of attitude outcomes, the attitudes evolved from four different subthemes, which included altered beliefs and values, increased willingness to help, reduced stigma and fear, reduced emotional energy, and increased intelligence [11, 30, 32, 34, 35].

Depending on the duration of the training, the participants improved their negative attitudes and had more willingness to help others in a more positive, patient, and responsible way. When they were eager to help those people who are in need, the more positive outcomes came out at the end of the supporting [11, 32, 34].

This review identified six interviews or questionnaires of MHFA training in a variety of settings. There were generally small to moderate improvements post training and could be seen up to 21 months throughout the results. The findings of the present study indicated that introducing the MHFA training course would benefit the general public, especially nursing students and healthcare workers. The nursing students had more feedback than the general public on the MHFA training course because nursing students had some related knowledge before related to this course.

This section will discuss whether this systematic review can be generalized or not. The papers were a little disparate in terms of their design, populations, exposures, and outcomes, which indicated it was difficult to compare outcomes across the quality studies or perform evidence synthesis. Therefore, the findings of this review should be generalized from the healthcare workers and then spread to the whole population worldwide.

There were a lot of common points in my study. Both Morgan et al. and my study have shared a number of key features, including improving knowledge, confidence, and intentions and altering behaviors after attending the training [36]. My review agreed with these positive experiences of Morgan et al. In terms of methods, this article was a systematic quantitative review, whereas my study was a systematic qualitative review [36]. Furthermore, this article focused on the data from the primary studies, while my review focused on the experiences of the participants [36]. Another related study showed that people who received MHFA improved in mental health literacy, but the changes in adolescents' mental health cannot be detected [37]. However, my study using the PICO tool covered slightly less comprehensively whether MHFA training is needed to be explored further in different countries. Adolescent MHFA is effective in increasing recognition and intention to assist suicidal peers similar to my outcome [38, 39].

The advantages and disadvantages of this systematic review are discussed in this paragraph. The search strategy was comprehensive. The reviewer searched the articles from different databases to ensure all the suitable papers were included. The secondary advantage is that it fully incorporates the subjects' experience of attending an MHFA training course. Another strength is the up-to-date primary studies. However, there was some bias in this review [23]. The articles are from four countries, and their full experience on the MHFA training course is not yet representative of the world's feel about the course [18]. Moreover, the review included a study of which population considered the instructors. This may increase the risk of bias [23]. This systematic qualitative review indicated that the MHFA training course could improve people's knowledge, practical skills, and alter their attitudes. This review can support the evidence who insist that the MHFA training course can reduce their stigma and improve confidence and knowledge.

This review demonstrated the positive effects of participants' experience on the MHFA training course. Although most of the included papers stated positive feedback on MHFA, there were some negative feedbacks from the participators. This may be because they had learned related knowledge before attending this course. What is more, some participants thought that the course should be more flexible and should contain interesting information to improve the efficiency of the course. In future research, the reviewer should analyze more articles such as e-learning MHFA and the YMHFA to undertake this course in-depth and find its meaning in improving people's life quality. For its population, more general participants need to be considered.

In short, different people had different experiences on the MHFA training course, which included both positive and negative outcomes. This was because there were different levels of knowledge of mental health illness; the higher level there was, the more requirements they would acquire. It is a significant outcome that the MHFA training course has different effects on different people, which means that the course still needs some improvements to satisfy a larger population in the future. The MHFA training course can be further developed into a specific course for old people, as their mental health issues differ from those of young people.

## 5. Conclusion

MHFA courses can provide participants with expertise in mental health counseling, improve their practical skills, and improve their attitude towards their patients.

## Data Availability

The datasets used and analyzed during the current study are available from the corresponding author upon reasonable request.

## Supplementary Materials

Supplementary Materials Appendix 1: Search strategy for one database used. Appendix 2: Table of include and exclude selection. Appendix 3: The differences among different tools of appraise qualitative research. Appendix 4: Critical appraisal forms. Appendix 5: Table of characteristics of included studies. Appendix 6: Analytical themes forms.
