# Supplementary material for: Impact of Mental Health First Aid Training Courses on Patients' Mental Health
Source: Comput Intell Neurosci. 2022 Sep 12;2022:4623869. doi: 10.1155/2022/4623869 (PMC9484930; doi:10.1155/2022/4623869)
Supplement: Supplementary Materials — Appendix 1: Search strategy for one database used. Appendix 2: Table of include and exclude selection. Appendix 3: The differences among different tools of appraise qualitative research. Appendix 4: Critical appraisal forms. Appendix 5: Table of characteristics of included studies. Appendix 6: Analytical themes forms. [file 4623869.f1.pdf]

Appendices

Appendix 1: Search strategy for one database used

Population	Phenomenon of interest	Context
Any (NOT "instructors" AND " facilitators")<br><br>**S3**	"experience*" OR "attitude*" OR "feeling*" OR "opinion*" OR "perception" OR "view*" OR "acceptability" OR "account*" "perspective*" OR "story" OR "stories"<br><br>**S1**	"mental health first aid" OR<br><br>"mental health illness" OR<br><br>"mental health issues" OR<br><br>"mental health problems" OR<br><br>"mental health disorders" OR "physiology disorders"<br>**S2**

Appendix 2: Table of include and exclude selection

	Include	Exclude
**Participants**	Any participant who experienced the MHFA training	Instructors who experienced the MHFA training
**Intervention**	Participants' experiences, views or accounts of the	Instructors' prospective views on the MHFA training

	MHFA training	
**Intervention setting**	Any workers in the public area, such as employees, nursing and midwifery students, health professionals.	Studies in instructors' who delivery the MHFA courses
**Study focus**	Experience, perceptions of the MHFA training and feedback	Focus on safety, effectiveness, impacts, evaluations, outcomes, effects, efficacy of the MHFA training or MHFA e-learning
**Outcome**	Any	N/A
**Study**	Primary qualitative studies including phenomenological, grounded theory, ethnography, action research, mix-methods studies with in-depth qualitative part	Quantitative studies, quantitative findings from mix-methods studies, free text boxes from quantitative surveys
**Time period**	2005 to 2019	Before 2005
**Publication**	Peer-reviewed published primary studies, theses, dissertations, research reports	Policy documents, conference abstracts, systematic reviews
**Language**	Papers written in English language	Papers not written in English

Appendix 3: The difference among different tools of appraise qualitative research

Appraisal Tool	Country and Discipline	Purpose(s)	Content and Details	Strengths and Criticisms
CASP Qualitative Checklist, 2016<br>Critical Appraisal Skills Programme (CASP) 10 Questions to help you make sense of qualitative research: Milton Keynes Primary Care Trust, 2002.	United Kingdom<br>N/A	1. Appraisal in a qualitative evidence synthesis<br>2. Reporting standards for qualitative papers<br>3. Educational tool for learners	10 checklist questions answered with a yes, no or can't tell<br>• Screening Questions (2): Aims of study and appropriateness of qualitative methodology to aims<br>• Appraisal Questions (8): Research design, recruitment strategy, data collection, reflexivity-related issues, ethical issues, rigor of data analysis, and the reporting and value of findings.<br>Press releases are available containing a discussion on quality criteria for qualitative research.	Strengths<br>• Easy to understand and administer.<br>• Easy to use as an educational tool for learners and novice researchers.<br>• Most commonly used appraisal tool in the quality appraisal process of qualitative evidence syntheses.<br>Criticisms<br>• Weaker in evaluation of methodological quality compared to other appraisal tools (Hannes, Lockwood, & Pearson, 2010).<br>• Adaptations of the tool are time-consuming to use (Campbell et al., 2003)<br>• Favors studies with better methodological quality but may make weaker contributions to field (Dixon-Woods et al., 2007)
COREQ<br>Tong, Sainsbury, and Craig (2007)	Australia,<br>Medicine	1. Reporting standards for qualitative papers<br>2. Appraisal in a qualitative evidence synthesis	32 structured statements in 3 domains:<br>• "Research Team and Reflexivity" (8): Researcher's personal characteristics and relationship with participants<br>• "Study Design" (15): Theoretical framework, sampling strategy, study setting and data collection procedures<br>• "Analysis of Findings" (9): Number of data coders, type of software used and method of reporting findings<br>Supplementary document published with Cochrane exploring issues with COREQ and quality for qualitative research.	Strengths<br>• Widely used and endorsed by many international journals.<br>• Developed through a systematic search and review of previously developed appraisal tools.<br>Criticisms<br>• Lack of emphasis on influence of philosophy on qualitative inquiry.<br>• Only applicable to studies that employ focus groups and interviews as data collection methods.<br>• Lack of guidelines to evaluate studies that use non-traditional data collection methods, or a combination of traditional and non-traditional methods.
ETQS<br>Long and Godfrey (2004)	United Kingdom,<br>Sociology	1. Appraisal in a qualitative evidence synthesis	38 unstructured questions under 4 themes:<br>• "Phenomenon Studied and Context" (17): Theoretical framework, setting, sample and outcomes<br>• "Ethics" (3): Approval from a research ethics board, informed consent and reporting on ethical issues<br>• "Data Collection, Analysis and Potential Research Bias" (10): Data collection process, analysis procedures and research bias<br>• "Policy and Practice Implications" (4): Policy and practice implications of study findings<br>Evaluative Abstract (4): Summary of a study's essential details and potential value (bibliographic details, key findings and summary of its strengths).	Strengths<br>• Very comprehensive.<br>• Evaluative abstract serves as a summary of the study.<br>Criticisms<br>• Lack of emphasis on philosophy and its congruity with methodology and methods.<br>• Requires qualitative expert use.<br>• More time-consuming than other appraisal tools.
JBI<br>Lockwood, Munn, and Porritt (2015)	Australia,<br>Medicine	1. Appraisal in a qualitative evidence synthesis	10 checklist questions answered with a yes, no, unclear or not applicable:<br>• Congruity (5): Congruity between philosophical perspective, methodology, research question and objectives, data collection methods, data analysis and interpretation of results<br>• Reflexivity (2): Research's reflexivity and influence on the data collection and analysis procedures<br>• Voice of Participants (1): Role of participants in the findings<br>• Ethics (1): Approval from an ethics body and other ethical issues<br>• Flow (1): Coherence between conclusions and interpretation of data<br>Supplementary document containing the methodology for development of JBI Critical Appraisal Checklist.<br>Supplementary document describing the development and use of Qualitative Assessment and Review Instrument.	Strengths<br>• Qualitative Assessment and Review Instrument (QARI) is an online software that streamlines the quality appraisal process using the JBI tool.<br>• Brevity and clarity permits its use by less experienced researchers.<br>• Better assessment of study details than other tools (Hannes, 2010).<br>• Short and easy to use.<br>Criticisms<br>• Main emphasis is on congruity between philosophy, methodology and methods.
Popay<br>Popay, Rogers, and Williams (1998)	United Kingdom,<br>Medicine	1. Appraisal in a qualitative evidence synthesis	Eight "markers":<br>• Findings illuminate subjective meaning<br>• Iterative nature of research methods<br>• Appropriateness of sample<br>• Adequate description of findings<br>• Triangulation between data sources<br>• Flow of data, examples, interpretation<br>• Claims of generalizability<br>No information on scoring of markers.	Strengths<br>• Seminal formulation of quality criteria for QES.<br>• Highlights the epistemological and ontological differences between qualitative and quantitative research.<br>Criticisms<br>• Lack of information on the tool's development methodology.<br>• Quality criteria have evolved and expanded since this work.<br>• No detailed guidelines on how to use and score this guide in the quality appraisal process making it difficult to report quality in a QES.
QF<br>Spencer, Ritchie, Lewis, and Dillon (2003)	United Kingdom,<br>Policy	1. Appraisal in a qualitative evidence synthesis<br>2. Primary purpose is for use in evaluative and policy research	18 structured appraisal questions with 86 quality indicators categorized under 9 themes<br>• Findings: Scope, explanation, contribution and credibility of findings<br>• Design: Defensibility of design<br>• Sample: Defensibility of sample<br>• Data Collection: Data collection process<br>• Analysis: Context and rigor of data analysis procedures<br>• Reporting: Link between data, interpretations and conclusions<br>• Reflexivity & Neutrality: Clarity of assumptions, perspectives and values<br>• Ethics: Evidence of attention to ethics<br>• Auditability: Adequate documentation of the research process<br>Each principle is associated with "quality indicators" that focuses on specific issues.	Strengths<br>• Transparent guidelines and development process.<br>• Very comprehensive.<br>Criticisms<br>• Very long and time-consuming.<br>• Lower reliability between appraisers than CASP (Dixon-Woods et al, 2007).<br>• Applies to only traditional data collection methods (i.e., interviews, documents, focus groups).<br>• Lack of guidelines on how to apply to distinct qualitative methodologies.
SRQR<br>O'Brien, Harris, Beckman, Reed, and Cook (2014)	United States,<br>Academic Medicine	1. Improve transparency of all aspects of qualitative research<br>2. Provide clear standards for reporting qualitative research<br>3. Assist the review process of evaluating a manuscript for potential publication<br>4. Appraisal in a qualitative evidence synthesis	21 structured statements into five themes:<br>• "Title and Abstract" (2): Concise description and summary<br>• "Methods" (11): Qualitative approach, reflexivity, context, sampling, ethical issues, data collection methods and instruments, units of study, data processing and analysis and techniques to enhance trustworthiness<br>• "Results/Findings" (2): Synthesis, interpretation and connections to data<br>• "Discussion" (2): Incorporation of prior work, implications on the field, transferability and limitations of study<br>Supplementary document containing examples of applying criteria is available.	Strengths<br>• Very transparent in purpose and development of appraisal tool.<br>• Developed through a systematic search and review of previously developed appraisal tools.<br>• Claims to consider all qualitative research methodologies equally.<br>Criticisms<br>• Nascent appraisal tool.<br>• Whether it truly considers all qualitative methodologies equally, as suggested, is unestablished.
Walsh<br>Walsh and Downe (2006)	United Kingdom,<br>Medicine	1. Appraisal in a qualitative evidence synthesis<br>2. Primary purpose is for use in rapid reviews in policy	12 "essential criteria" statements under eight stages of research process:<br>• Scope and Purpose: Study rationale, research question and existing literature<br>• Design: Study design and data collection strategies.<br>• Sampling Strategy: Eligibility criteria and appropriateness for methodology<br>• Analysis: Coherence of analytical approach with sampling and design<br>• Interpretation: Adequate description of context, audit trail and coherence between data and conclusions<br>• Reflexivity: Sufficient description of how researcher may have influenced the findings<br>• Ethical Dimensions: Researchers demonstrated concern about risk to human participants<br>• Relevance and Transferability: Extent to which findings can be applied to different contexts<br>These criteria are to be used "imaginatively" instead of prescriptively, and therefore, rating of criteria is not recommended.	Strengths<br>• Use of "berry-picking" approach to find existing appraisal tools provides a real-world perspective where finding one tool online leads to finding another tool. This approach mimics how appraisers would locate tools when appraising the quality of a qualitative paper.<br>Criticisms<br>• Berry-picking approach is non-systematic and therefore not representative of the literature.<br>• Limited information on details on development methodology.<br>• Use of criteria "imaginatively" instead of prescriptively makes it difficult to report quality in a QES.

Note. QES = qualitative evidence syntheses.

Appendix 4: Critical appraisal forms

Author<br><br>Year	1. Is there congruity between the stated philosophical perspective and the research methodology?	2. Is there congruity between the research methodology and the research question or objectives?	3. Is there congruity between the research methodology and the methods used to collect data?	4. Is there congruity between the research methodology and the representation and analysis of data?	5. Is there congruity between the research methodology and the interpretation of results?	6. Is there a statement locating the researcher culturally or theoretically?	7. Is the influence of the researcher on the research, and vice-versa, addressed?	8.Are participants, and their voices, adequately represented?	9. Is the research ethical according to current criteria or, for recent studies, and is there evidence of ethical approval by an appropriate body?	10. Do the conclusions drawn in the research report flow from the analysis, or interpretation, of the data?	11. Comments
Hung et al., 2019	Yes	Yes	Yes	Yes	Yes	Not clear	Yes	Yes	Yes	Yes	The paper's congruity is high, although the author's culture did not mention in the paper, it did not affect the results of the study and can be included.<br><br>Overall: included
Jorm et al., 2005	Yes	Yes	Yes	Yes	Yes	Not clear	Yes	Yes	No	Yes	The paper's congruity is high, although the author's culture and participants' ethics were not mentioned in the paper, it will not affect the results of the study and can be included.

											Overall: included
Kelly and Birks, 2017	Yes	Yes	Yes	Yes	Yes	Not clear	Yes	Yes	Yes	Yes	The paper's congruity is high, although the author's culture did not mention in the paper, it did not affect the results of the study and can be included.<br><br>Overall: included
Luckstед et al., 2015	Yes	Yes	Yes	Yes	Yes	Not clear	Yes	Yes	No	Yes	The paper's congruity is high, although the author's culture and participants' ethics were not mentioned in the paper, it will not affect the results of the study and can be included.<br><br>Overall: included
Rodgers et al., 2019	Yes	Yes	Yes	Yes	Yes	Not clear	Yes	Yes	No	Yes	The paper's congruity is high, although the author's culture and participants' ethics were not mentioned in the paper, it will not affect the results of the

											study and can be included.<br><br>Overall: included
Svensson et al., 2015	Yes	Yes	Yes	Yes	Yes	Not clear	Yes	Yes	Yes	Yes	The paper's congruity is high, although the author's culture did not mention in the paper, it did not affect the results of the study and can be included.<br><br>Overall: included

Appendix 5: Table of characteristics of included studies

Author Year	Methodology	Method	Phenomena of interest	Setting	Geographical	Cultural	Participants	Data analysis	Author's conclusions	Comments	Complete	Findings	Illustration form Publication (page numbers)	Evidence
1. Hung et al., 2019	qualitative descriptive method	semi-structured interviews	To explore nursing students' experience of attending the MHFA training program	university	Hong Kong, China	full-time Bachelor's nursing degree students	25 nursing students who have completed the 12-hour MHFA training course	Thematic analysis	The results show that MHFA training courses can improve the psychological practice and experience of nursing students, which can be integrated into teaching.	Nursing students also attend similar classes prior to the MHFA course, which may lead to a better understanding and acceptance.	Yes	Reinforced knowledge and understanding; Enhanced techniques and skills; Rectified beliefs and values; Improved self-awareness of personal mental health status; Enhanced sense of achievement and satisfaction	*Collegian.* 26 (5)<br><br>Pp.534-540	Unequivocal
2. Jorm et al.,	qualitative	questionnair	To explore participants'	not	Australia	not	94 Participants,	Thematic	Qualitative data confirm that most MHFA training members of the public then provide	The respondent rate is only 78%, which may influence the outcome of	Yes	Achieved better results; Experienced positive effects in increasing	*BMC psychiatry*,	Credibl

2005	descriptive method	e	experience of attending the MHFA training program	clear		clear	who have complete MHFA course after 19-21 months later	analysis	support to people with mental health problems, which usually has a positive effect.	the feedback.		empathy, confidence and managing crises better; experienced positive effects; very useful and very eager to extend it	*5*(1), p.43.	e
3. Kelly and Birks, 2017	qualitative descriptive method	online survey	To explore the experience of undergraduate nursing and midwifery students who completed a tailored 13-h MHFA course	university	Australia	undergraduate nursing and midwifery students	66 students, the majority of whom were female and aged 17-55 years	Content analysis	The data show that the program's application and impact on completion have exceeded student expectations, and responses to open-ended questions indicate widespread support for a nationwide program for beginning nursing and midwifery students.	Limit determined in this study and implement suggestions will support MHFA course introduction of the school of nursing and midwifery in an ongoing and sustainable way, eventually made important contribution to the value of professional nursing and midwifery students	Yes	The results showed that the overwhelming majority of students (86 percent) thought the course was suitable for nursing and midwifery students, while 89 percent said they would recommend it to other college students.	*Collegian*, *24*(3), pp.275-280.	Unequivocal
4. Luckstead et al., 2015	qualitative descriptive method	semi-structured interview	To explore graduates' experience of attending MHFA traing	not clear	The USA	All attened at least some college and 10(50%) hold an advanced degree	20 interviewees, 15 were MHFA-USA instructors. 16(80%) are female, 14(70%) are White and 4(20%) African American	Coding analysis	As MHFA reaches more and more people around the world, it is crucial to understand its impact on mental health promotion and other outcomes -- on students, people in distress, wider organisations and communities -- using different survey methods. Given the widespread lack of mental health knowledge, the wide gap between the needs and help of the poor, the prejudice against people with mental health problems and the many costs of menatl health services, the need is enormous. MHFA is a promising public health and community resource, but its potential impact has not been fully tested.	This study was limited by the deterministic characteristics of its samples, especially the 75% of MHFA researchers who completed the 5-day instructor training and were able to impart mhfa-usa to others, and their satisfaction was higher than others.	Yes	Respondents said they gained the knowledge, skills and confidence to help people in need, had empathy for people with mental illness and developed a sense of responsibility and responsibility to reach out when needed. They gave different examples, such as using mhfa-usa skills among strangers, acquaintances, colleagues, clients and family, and helping themselves in various situations.	*International Journal of Mental Health Promotion*, *17*(3), pp.169-183.	Credible
5. Rodgers et al., 2019	mixed-methods study	semi-structured interviews	To explore university students' experience of MHFA training	university	Australia	university students	9 students who had completed the MHFA training programm at least 12-months prior and were part of a MHFA training	Thematic analysis	The qualitative results of this study demonstrate the role of MHFA training in the real world. Mental health knowledge, cognitive skills, and confidence gave participants a sense of mission in helping mental health patients. The complexities students experience include helping individuals with mental health problems and the benefits of MHFA in different situations. This study found that high confidence is related to algebraic behavior and plays an important role in helping behavior of interviewees.	Due to the small size of the study, the ability to detect differences in major outcome variables was inadequate. Collect data on each problem completed by all eligible participants; Failure to complete a particular problem can lead to bias. Recall bias is a limitation due to the length of time required to complete MHFA training.	Yes	Challenging experiences (personal investment in relationships; Emotional energy); Positive experiences (self-confidence; Stronger relationships and trust; Emotional intelligence	Advances in Mental Health. 10. pp1-23.	Unequivocal

6. Svensson et al., 2015	qualitative descriptive method	interview	To explore participants' experience of the MHFAprogram	employees from a diversity of organizations were represented	Sweden	Different occupational groups include employment agents, social workers, deacons, care workers and various health professionals	24 people (16 women, 8 men), most of the participants had previous experience from meeting persons with mental ill health, only a minority lacked such experience	Content analysis	For those who have had complex experiences with people with mental health problems, participation in the course seems to help boost their confidence and make them more willing to take action when meeting people with mental health problems. This seems to be professional and private are effective. Better understanding and stronger preparation for action may contribute to earlier access to help, and may prevent further illness, as a result of specific recommendations, a decline in confidence in action, and may also contribute to the work of professionals who often encounter people in crisis. Compared with the general population, the sample may require participants to have a more positive attitude and early knowledge of mental health problems, for their part, MHFA training value may be worth further exploration in Sweden.	The study sample is limited, although represent the many different professional and organizational property. As mentioned earlier, the sample composition may contain a group with a more positive attitude and knowledge of mental health issues, which may introduce bias	Yes	Raise awareness; Knowledge and understanding; Influence on attitudes and approaches; Toolbox and confidence; Feedback on content and layout; And specific examples of applied knowledge. The most important findings are the practical focus of the project and the results used, the increased confidence and propensity to take action after project involvement, and the importance of experienced mentors.	*Community mental health journal*, *51*(4), pp.497-503.	Credible

Appendix 6: Analytical themes forms

Analytical themes	Description themes	Study	Source
1. Improved individuals' MHFA knowledge and practical ability	1.1 Enhanced knowledge and understanding	1.1 (Hung et al., 2019; Jorm et al., 2005; Kelly and Birks, 2017; Lucksted et al., 2015; Rodgers et al., 2019; Svensson et al., 2015)	Participants' quote and author's conclusion
	1.2 Positive effects and feedback	1.2 (Hung et al., 2019; Jorm et al., 2005; Kelly and Birks, 2017; Lucksted et al., 2015; Rodgers et al., 2019; Svensson et al., 2015)	Participants' quote and author's conclusion
	1.3 Improved mental health techniques and skills	1.3 (Hung et al., 2019; Jorm et al., 2005; Kelly and Birks, 2017; Lucksted et al., 2015; Rodgers et al., 2019)	Participants' quote and author's conclusion
	1.4 Improved confidence	1.4 (Jorm et al., 2005; Lucksted et al., 2015; Rodgers et al., 2019; Svensson et al., 2015).	Participants' quote and author's conclusion
	1.5 Increased self-awareness of individuals' mental health conditions	1.5 (Hung et al., 2019; Jorm et al., 2005; Lucksted et al., 2015)	Participants' quote and author's conclusion

	1.6 Increased the sense of accomplishment and satisfaction	1.6 (Hung et al., 2019; Jorm et al., 2005)	Participants' quote and author's conclusion
2. Improved individuals' attitude	2.1 Altered beliefs and values	2.1 (Hung et al., 2019; Jorm et al., 2005; Lucksted et al., 2015; Svensson et al., 2015)	Participants' quote and author's conclusion
	2.2 Increased willingness to help	2.2 (Hung et al., 2019; Jorm et al., 2005; Lucksted et al., 2015)	Participants' quote and author's conclusion
	2.3 Reduced stigma and fear	2.3 (Lucksted et al., 2015; Rodgers et al., 2019)	Participants' quote and author's conclusion
	2.4 Reduced emotional energy and increased intelligence	2.4 (Rodgers et al., 2019)	Participants' quote and author's conclusion
